# Acute massive gastric distension in acute pancreatitis

**DOI:** 10.11604/pamj.2019.33.8.18769

**Published:** 2019-05-07

**Authors:** Susanta Meher, Nishant Debta

**Affiliations:** 1 Department of Surgery, Vikash Multispeciality Hospital, Bargarh, Odisha, India; 2 Department of Medicine, Vikash Multispeciality Hospital, Bargarh, Odisha, India

**Keywords:** Acute pancreatitis, massive gastric distension, perforation peritonitis

## Image in medicine

A 43 year old male patient presented to our emergency department with complain of severe abdominal pain for five days associated with fever, vomiting and non-passage of flatus and stool. The pain started in the epigastric region which became diffused in the subsequent days. Last two days patient had history of breathlessness and decreased urination. On examination patient was looking toxic with tachycardia (124/min) and tachypnea (26/min) with rapid and shallow breathing. Abdominal examination revealed a rigid abdomen with tympanic notes with absent bowel sound. Laboratory investigations showed leukocytosis (15,000/cmm) with raised serum urea (235mg/dl) and creatinine (5.3mg/dl). There was no gas under diaphragm on abdominal X-Ray (A). With high degree of suspicion of perforation peritonitis, a diagnostic peritoneal tapping was done from right lower abdomen and altered colored bilious fluid was aspirated. Patient was resuscitated and planned for emergency laparotomy, which revealed a massively distended stomach covering almost whole of the abdomen (B, C). Duodenum and proximal part of jejunum was also dilated without any mechanical obstruction. Pancreas appeared edematous and bulky with saponification near the root of mesentery. Post-operative serum amylase (494u/l) and lipase (211u/l) as well as peritoneal fluid amylase (826u/l) levels were high, suggestive of acute edematous pancreatitis. Peritoneal tapping is sometime used to detect sealed perforation in cases with signs of peritonitis but without evidence of free gas on radiological investigation. Massive gastric distension in acute pancreatitis is very rare, however peritoneal tapping in such conditions can give a false impression of free fluid (bilious) in the peritoneal cavity.

**Figure 1:**
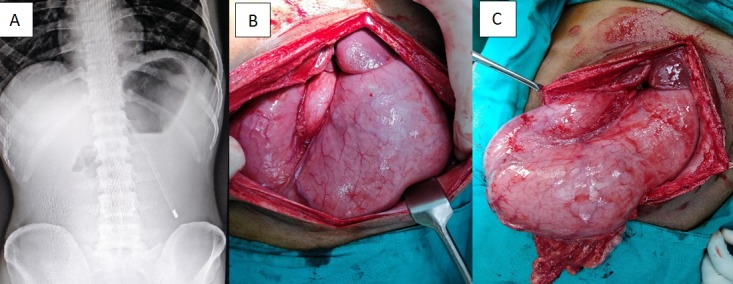
(A) X-Ray of the abdomen showing ground glass appearance with a single air-fluid level without any evidence of gas under both domes of diaphragm; (B) massively distended stomach covering almost all the quadrants of the abdomen; (C) massively distended stomach with no evidence of any peritoneal fluid collection

